# Differential role of a persistent seed bank for genetic variation in early vs. late successional stages

**DOI:** 10.1371/journal.pone.0209840

**Published:** 2018-12-26

**Authors:** Benjamin Schulz, Walter Durka, Jiří Danihelka, Rolf Lutz Eckstein

**Affiliations:** 1 Institute of Landscape Ecology and Resource Management, Justus Liebig University Giessen, Giessen, Germany; 2 Department of Community Ecology (BZF), Helmholtz Centre for Environmental Research–UFZ, Halle (Saale), Germany; 3 German Centre for Integrative Biodiversity Research (iDiv) Halle-Jena-Leipzig, Leipzig, Germany; 4 Department of Botany and Zoology, Masaryk University, Brno, Czech Republic; 5 Institute of Botany, The Czech Academy of Sciences, Průhonice, Czech Republic; 6 Department of Environmental and Life Sciences–Biology, Karlstad University, Karlstad, Sweden; Brigham Young University, UNITED STATES

## Abstract

Persistent seed banks are predicted to have an important impact on population genetic processes by increasing effective population size and storing past genetic diversity. Accordingly, persistent seed banks may buffer genetic effects of disturbance, fragmentation and/or selection. However, empirical studies surveying the relationship between aboveground and seed bank genetics under changing environments are scarce. Here, we compared genetic variation of aboveground and seed bank cohorts in 15 populations of the partially cleistogamous *Viola elatior* in two contrasting early and late successional habitats characterized by strong differences in light-availability and declining population size. Using AFLP markers, we found significantly higher aboveground than seed bank genetic diversity in early successional meadow but not in late successional woodland habitats. Moreover, individually, three of eight woodland populations even showed higher seed bank than aboveground diversity. Genetic differentiation among populations was very strong (ф_ST_ = 0.8), but overall no significant differentiation could be detected between above ground and seed bank cohorts. Small scale spatial genetic structure was generally pronounced but was much stronger in meadow (*Sp*-statistic: aboveground: 0.60, seed bank: 0.32) than in woodland habitats (aboveground: 0.11; seed bank: 0.03). Our findings indicate that relative seed bank diversity (i.e. compared to aboveground diversity) increases with ongoing succession and despite decreasing population size. As corroborated by markedly lower small-scale genetic structure in late successional habitats, we suggest that the observed changes in relative seed bank diversity are driven by an increase of outcrossing rates. Persistent seed banks in *Viola elatior* hence will counteract effects of drift and selection, and assure a higher chance for the species’ long term persistence, particularly maintaining genetic variation in declining populations of late successional habitats and thus enhancing success rates of population recovery after disturbance events.

## Introduction

Instead of relying solely on the spatial dispersal of their seeds, many plant species have developed the ability to disperse their offspring in time by accumulating long-lived, dormant seeds of several years in the soil or in aerial reservoirs. Such persistent seed banks are a common feature of plants to counteract the consequences of environmental or demographic stochasticity that can be found across a wide range of life history types, habitats and climate zones [1,2]. Particularly for rare species with disjunct populations or for species from highly dynamic or disturbed habitats, persistent seed banks may be crucial for population dynamics and stability, and potentially are capable of replacing standing individuals after bottlenecks or extinction events [3,4].

Theory predicts that, beyond affecting demography, seed banks could have an important impact on population genetic processes as they may consist of progeny produced by several and variable above-ground generations under varying selection regimes [5,6]. Consequently, persistent seed banks may increase effective population size [7,8] and store even more genetic diversity than present in the aboveground populations [5]. Thereby, they could buffer deleterious effects of random genetic drift and weaken patterns of genetic population structuring [5,9]. In addition, seed banks may enable gene flow from past generations stored in the soil, maintain genes in populations through periods in which they are selected against [6], and thus slow down adaptation processes and damp out directional selection in response to environmental fluctuations.

A number of studies have been carried out to empirically test the ecological and evolutionary impact of long-lived seed reservoirs. Some of these indicated that persistent seed banks might indeed increase effective population size, both in annuals [10,11] and perennials [12]. However, so far, it was impossible to conclusively confirm the potential of seed banks to accumulate genetic diversity and to serve as a genetic memory that might influence the evolutionary fate of populations [13]. Some studies found higher genetic diversity in seed banks [9,14], whereas most others showed higher diversity in standing plants (e.g. [6,15,16]) or no significant differences (e.g. [10–12,17,18]).

To shed some more light on this topic, Honnay et al. [19] conducted a meta-analysis using 13 studies that compared the genetic diversity of seed banks and aboveground populations. Interestingly, whereas levels of heterozygosity and percentage of polymorphic loci appeared to be similar in the two groups, allelic richness was significantly higher in the seed bank. Honnay et al. [19] concluded that differences in allelic richness are mainly driven by rare alleles and that selection might act as filter on seed bank alleles, preventing some of them to be established in standing plants. Moreover, the analysis showed significantly higher inbreeding and more homozygotes in the seed bank, substantiating the results of earlier studies, showing a gradual increase of heterozygosity towards the adult stage [16,20,21]. As discussed by Vitalis et al. [8], this pattern is most likely explained as an effect of selection that progressively eliminates less fit homozygotes in the course of seed germination and recruitment [16,22].

Overall, the study of Honnay et al. [19] thus gave no evidence that high levels of genetic diversity are accumulated in long-lived seed reservoirs. Instead, any difference in the genetic composition between seed bank and aboveground population may rather be the result of local selection than a buffering effect of stored seeds [8,19]. Consequently, the authors discouraged to continue surveying the genetic diversity of the two cohorts, unless this is performed under different selection regimes, in order to compare the outcome of the selection process [19]. However, until today the aspect of selection has been largely omitted in empirical research on seed bank genetics. To our knowledge, only three studies have yet compared seed bank and aboveground plants in contrasting environments and thus potentially under different selection regimes [11,23,24]. None of these showed clear differences in genetic diversity, neither between seed bank and aboveground plants nor among habitats. This might partially be attributed to comparatively small data sets and hence idiosyncratic characteristics of single populations.

To fill this gap in current knowledge, here we compared the genetic variation of seed bank and aboveground cohorts in the perennial flood plain species *Viola elatior* in two habitats representing the endpoints of a management-driven successional gradient, open grassland and alluvial forests fringes. The study species is a weak competitor for light [25]. Consequently, with increasing succession to closed forests, population sizes gradually decline and the species finally disappears from the aboveground vegetation [26]. Light availability is a strong environmental cue that influences an array of biotic and abiotic parameters (e.g. competition, water availability or temperature) and went along with epigenetic population differentiation in this species [27]. Therefore, the surveyed successional states appear well suited to study the outcome of local selection on the interplay of seed bank and aboveground genetics. Besides levels of standing genetic variation *per se*, also small-scale genetic structure may reveal patterns that give insight into spatio-temporal gene flow. We conducted a multi population study with 15 localities, covering two main distribution areas of *V*. *elatior* in Germany and the Czech Republic. Using amplified fragment length polymorphisms (AFLP) we asked the following questions: (1) Can the persistent seed bank of *V*. *elatior* maintain genetic diversity in different successional habitats with different selection regimes? (2) Do contrasting habitat types impact the small-scale spatial genetic structure of the seed bank and/or the aboveground plants?

## Materials and methods

Permissions for field work and collection of plant tissues were issued by the Regional council Darmstadt (Regierungspräsidium Darmstadt) for the populations in the Rhine region (Germany) and by the South Moravian Regional Authority, Brno, Department of Environment (Krajský úřad Jihomoravského kraje, Brno, odbor životního prostředí) for populations in the Thaya/Morava region (Czech Republic).

### Study species

*Viola elatior* (Violaceae) is a perennial iteroparous hemicryptophyte belonging to the section *Viola*, subsect. *Rostratae* [26]. The species’ distribution roughly covers the submeridional and temperate zone of western Eurasia ranging from the Parisian basin to southern Siberia and Central Asia [28]. In its core-area with summer-warm continental climates, *V*. *elatior* grows in steppe and forest-steppe vegetation, whereas in Central Europe, i.e. towards the western border of its distribution, the species is confined to large river corridors [26,29]. Here it becomes increasingly rare and occurs in different successional floodplain habitats ranging from managed open floodplain meadows to alluvial woodland fringes and gaps [26,30]. Populations are usually small, varying between tens and hundreds of individuals [26].

*Viola elatior* has an octoploid genome (2n = 40) and exhibits a mixed mating system with potentially cross-pollinated chasmogamous and obligatory self-pollinated cleistogamous flowers (for simplification hereafter just called “outcrossing” and “selfed” flowers, respectively). Whereas outcrossing flowers are only produced in the beginning of the growing season, i.e. May to early June, selfed flowers may develop from June to late October. Both flower types produce capsules with approximately equal numbers of seeds (~30). Nonetheless, seed production through selfed flowers strongly prevails, resulting in a very high overall selfing rate. In common garden experiments only around 4% of total capsule production resulted from outcrossing flowers ([30], Schulz, unpublished data).

As many other violets, *V*. *elatior* builds up persistent soil seed banks. Hölzel & Otte [3] found maximum seed densities of up to 2660 germinable seeds/m^2^ under a densely populated floodplain meadow, with more than 80% of all seeds in the upper 5 cm of the soil layer. Under strongly fluctuating conditions of floodplain habitats, the seed bank seems to be an important part of the species’ life strategy, which is illustrated by various reports about sudden emergence of plants in the course of disturbance events after long-term absence from the aboveground vegetation ([26] and reference therein).

### Study regions

The study was conducted in two regions that represent strongholds of *Viola elatior* in Europe: The Upper Rhine floodplain south of the city Frankfurt am Main, Germany, and the Thaya/Morava floodplain around the town of Břeclav, the Czech Republic. In the densely populated and highly agriculturally influenced Upper Rhine area, the species occurs in nature reserves with a high share of open floodplain meadows that are regularly managed by mowing or grazing and thus provide high proportions of suitable early- and mid-successional habitats. In contrast, large parts of the Thaya/Morava floodplain are less influenced by settlements and intense land-use, and the landscape is characterized by a high share of alluvial forests and extensively managed patches of floodplain meadows. Here, populations of *V*. *elatior* are more widely scattered and mostly occur in late-successional habitats in gaps within forest stands or along forest fringes [31].

### Sampling design

In both regions we surveyed stands (hereafter called populations) from each of the two extremes of the species’ environmental range, i.e. sunny floodplain meadows and shady alluvial woodland fringes. First, all known sites in the two regions inhabiting *V*. *elatior* were inspected and visually classified according to their light environment. The light environment of each population (Table 1) was measured indirectly with hemispherical photography as mean daily percentages of transmitted total photosynthetic active radiation (for details see [27]). Then, representative populations of both habitat types (Table 1) were selected and aboveground and seed bank samples were collected between May and June, i.e. after the spring germination peak and before the new seed rain.

**Table 1:** Overview of surveyed populations of *Viola elatior*.

Population	Region	Latitude	Longitude	AG sample number	SB sample number *	Populated grid cells (m^2^)	Habitat type	Mean transmitted PAR ± SD (%)
RM1	Upper Rhine (Ger)	49°50'16''N	8°24'00''E	21	20 (11)	14	meadow	95.9 ± 0.2
RM2	Upper Rhine (Ger)	49°50'01"N	8°25'32"E	19	20 (12)	~1400	meadow	87.7 ± 6.5
RM3	Upper Rhine (Ger)	49°49'49"N	8°28'03"E	22	12 (8)	159	meadow	73.8 ± 8.3
RM4	Upper Rhine (Ger)	49°36'08"N	8°26'50"E	22	19 (7)	170	meadow	80.5 ± 14.7
RW1	Upper Rhine (Ger)	49°48'50"N	8°24'57"E	23	21 (10)	158	woodland	23.6 ± 14.2
RW2	Upper Rhine (Ger)	49°35'49"N	8°26'50"E	20	20 (12)	31	woodland	12.5 ± 3.3
RW3	Upper Rhine (Ger)	49°35'44"N	8°25'55"E	22	19 (10)	144	woodland	16.5 ± 9.4
TM1	Thaya/Morava (Cz)	48°45'50"N	16°51'57"E	21	23 (14)	32	meadow	48.3 ± 21.2
TM2	Thaya/Morava (Cz)	48°46'52"N	16°51'48"E	21	23 (13)	~1200	meadow	83.0 ± 4.7
TM3	Thaya/Morava (Cz)	48°48'51"N	16°49'53"E	21	22 (14)	160	meadow	60.1 ± 14.9
TW1	Thaya/Morava (Cz)	48°49'00"N	16°27'08"E	23	23 (14)	131	woodland	22.4 ± 11.7
TW2	Thaya/Morava (Cz)	48°49'25"N	16°46'26"E	23	23 (14)	59	woodland	25.3 ± 20.2
TW3	Thaya/Morava (Cz)	48°49'01"N	16°47'41"E	23	22 (13)	34	woodland	14.8 ± 7.4
TW4	Thaya/Morava (Cz)	48°38'21"N	16°57'19"E	22	21 (12)	56	woodland	13.1 ± 3.2
TW5	Thaya/Morava (Cz)	48°58'30"N	17°23'09"E	21	23 (15)	59	woodland	30.6 ± 13.4

* Numbers in brackets indicate germination trays that contributed with one or more emerged seedlings to the SB samples, respectively;

Ger—Germany; Cz—Czech Republic; AG—aboveground; SB—seed bank; PAR—photosynthetic active radiation.

Due to limited numbers of appropriate populations in the different habitat types with more than 20 aboveground individuals, we chose four meadow and three woodland sites in the Rhine region and three meadow and five woodland sites in the Thaya/Morava region (Table 1). Distances between populations ranged from 0.5 to 27 km in the Rhine region and from 1.6 to 70 km in the Thaya/Morava region.

To capture a maximum of allelic diversity and to detect potential spatial genetic structure within populations, we adopted a grid-based randomized sampling protocol. Therefore, in each population the presence of *Viola elatior* was mapped on a 1 m grid. For aboveground cohort sampling, we randomly selected 19–23 populated grid cells at each site and collected young, undamaged leaves from one plant per cell. Only for RM1, two to three individuals were sampled in 6 of the grid cells as the total number of populated grid cells did not exceed 14. Samples were immediately cooled to below 10°C, stored at -25°C and then freeze-dried for 48 h.

For seed bank cohort sampling, we applied the seedling emergence method. Using a soil corer of 5 cm in diameter and 4 cm in depth, we took five soil samples each in 30 randomly selected grid cells per population. The 150 soil samples represented 0.3 m^2^ of soil surface and 11.8 l of soil volume. As previous studies showed that *V*. *elatior* seeds are strongly dormant during summer, with almost no germination between May and September [26], the soil cores were stored dry and dark until autumn. To reduce soil volume and optimize germination conditions, we concentrated the soil samples by washing through two sieves with mesh sizes of 2.4 and 0.7 mm [32]. Seeds of *V*. *elatior* (diameter: 1.2–2.2 mm) thus accumulated in the middle fraction, whereas larger and smaller soil components were removed. Afterwards, the concentrated soil samples were spread in a ~0.5 cm thick layer on sterilized potting soil in 18 cm × 28 cm styrofoam trays. Due to logistic reasons, soil concentrates of two proximate grid cells were always pooled in one tray, respectively, resulting in 15 soil pools per population. For stratification, the trays were placed outside to expose them to natural conditions starting in late November. From the following spring to autumn the trays were watered regularly, and germinated *V*. *elatior* seedlings were identified and carefully transferred to individual pots once every month. The potted seedlings were grown in a greenhouse until they reached the four-leaf stage and were then harvested, stored at -25°C and finally freeze-dried for 48 h. Depending on germination success, 12–23 seed bank individuals per population were chosen for genetic analyses. To obtain a comparable spatial sample resolution as for aboveground individuals, whenever possible only one to two samples per germination tray were selected (Table 1).

In summary, for each of the four region × habitat combinations we sampled between 3 and 5 populations, consisting of 19–23 aboveground cohort individuals and 12–23 seed bank cohort individuals, respectively (Table 1).

### AFLP genotyping

We investigated a total of 324 aboveground and 311 seed bank samples with amplified fragment length polymorphism (AFLP). Total genomic DNA was extracted from dried leaf tissue using the DNeasy 96 Plant extraction kit (QIAGEN). AFLP methodology followed Kloss et al. [33] and is described in detail in S1 Text. After an initial screening of 64 primer pairs, eight selective primer combinations (S1 Table) were chosen for AFLP analyses. Separation and visualization of fragments was done on an ABI 3130 capillary sequencer (Applied Biosystems, Foster City, USA) with Genescan 500(-250) LIZ internal size standard (Applied Biosystems). GENMAPPER version 3.7 (Applied Biosystems) was used to analyze the AFLP profiles. We binned fragments manually for all samples in one batch using a peak height threshold of 10 rfu. Then, peak height data were exported and for each fragment a specific peak height threshold was manually determined based on the peak height distribution which allowed scoring presence (1) and absence (0) of fragments. All loci that showed a monomorphic pattern or a deviation in only one individual were excluded from the data set to prevent biased parameter estimation. Overall error rate was 0.6%, based on 58 replicate samples (9%) that were repeated starting with DNA extraction.

### Data analysis

We analyzed the binary AFLP data using a band- or marker-based strategy, i.e. without calculating allele frequencies. We estimated genetic diversity as band richness (*Br*) and as percentage of polymorphic loci at the 5% level (*PLP)* using AFLPDIV 1.1 [34]. To account for the unequal sample size of aboveground and seed bank individuals (Table 1), a rarefaction-based approach was employed with a standardized sample size equal to the smallest sample population (i.e. n = 12). To evaluate the number of bands within populations that are private to either aboveground or seed bank cohorts within each population, we calculated private band richness (*PBr*) with rarefaction analyses according to Kalinowski [35] separately for each population using ADZE 1.0 [36] and applying the same standardized sample size of n = 12. Finally, we estimated the proportion of unique AFLP-phenotypes *P*_*u*_ = (eMLG-1)/(N_min_-1) for each cohort using the R package poppr [37], where eMLG is the expected number of multilocus genotypes based on rarefaction to the smallest sample size N_min_. Statistical analyses comprised linear mixed models (LMM) testing the effects of the fixed factors region, habitat and cohort, and the interaction between habitat and cohort, while population served as random factor. Visual checks of model residuals led us to logit-transform *Br*, *PLP* and *PBr*, whereas *P*_*u*_ was not transformed before final analyses. Statistical analyses were done using the package lme4 and analysis of deviance tables with Wald II chi^2^ tests using the package car in R version 3.3.3 [38]. Additionally, to improve comparability between populations and to address the relative diversity of seed bank in comparison to aboveground cohorts across regions, for all diversity parameters differences between cohorts were depicted using the natural-logarithmic response ratios (LnRR) as proposed by Goldberg and Scheiner [39]: $LnRR=Ln(\frac{PSB}{PAG})$, where *P*_*SB*_ is the mean value of seed bank cohorts and *P*_*AG*_ is the mean value of aboveground cohorts. Differences between cohorts were considered significant when the 95% confidence interval did not overlap with zero.

We performed non-hierarchical and hierarchical analyses of molecular variance (AMOVA) using ARLEQUIN 3.5.1.2 [40] to quantify genetic variation among populations (ф_ST_), among groups of populations (ф_CT_) and among populations within groups (ф_SC_). To test for differentiation between aboveground and seed bank within each population, pairwise ф_ST_ values were calculated among cohorts. Significance levels were determined after 9999 permutations. Clustering of individual samples was examined with principal component analysis (PCA) using the R package ADEGENET v1.4–2 [41].

To examine the small-scale spatial genetic structure of aboveground and seed bank cohorts within and between habitats we used spatial autocorrelation methods implemented in SPAGeDi v1.4 [42]. We chose distance limits of 4, 8, 12, 16 and 20 m to assure a sufficient number of individual pairs per distance class. For seed bank cohorts, only samples originating from soil pools with a maximum distance of 4 m between the two soil sampling sites (see seed bank sampling strategy above) were considered, using the corresponding midpoint coordinates for the analyses, respectively. Thus, potential biases from the actual coordinates of the soil samples lie within the chosen distance classes, respectively. To construct spatial autocorrelograms, pairwise kinship coefficients (*F*_*ij*_) for dominant markers [43] were calculated assuming an inbreeding coefficient of 0.5. Using higher inbreeding coefficients of up to 0.9 in additional trials had little effect on the results and did not change the general conclusions. Significance of mean *F*_*ij*_ per distance class was tested with 9999 permutations of multilocus genotypes. We quantified small-scale spatial genetic structure for each population using restricted regression analyses (0–20 m) and calculating the *Sp* statistic, representing the rate of decrease in pairwise kinship with distance [44]. *Sp* was estimated as -*b*_log_/(1-*F*_(1)_), where *b*_log_ is the regression slope of mean *F*_*ij*_ on log geographic distance and *F*_(1)_ is the mean *F*_*ij*_ of the first distance class. To compare autocorrelation patterns, we furthermore pooled populations according to habitats and regions and tested for heterogeneous autocorrelation with heterogeneity tests for multiple populations subsets [45] using GenAlEx 6.5 [46] and applying the same distance classes as above. Number of permutations and bootstraps were set to 9999, respectively. Following Banks and Peakall [47] significance of the Heterogeneity Test is declared when *p*<0.01.

## Results

### Seedling emergence rates

Total numbers of seedlings that emerged from the concentrated soil cores strongly varied between populations (S2 Table) ranging from 12 in RM3 (40 seedlings/m^2^) to 206 in TW2 (687.7 seedlings/m^2^). Overall, mean values of germinated seedlings differed significantly between regions (Rhine = 24.9±9.4 and Thaya/Morava = 104.8±48.5; *t*-test, *p* = 0.003) but not between habitat types (meadow = 45.1±34.7 and woodland = 87±59.4; *t*-test, *p* = 0.15).

### Genetic diversity

**Fig 1:**
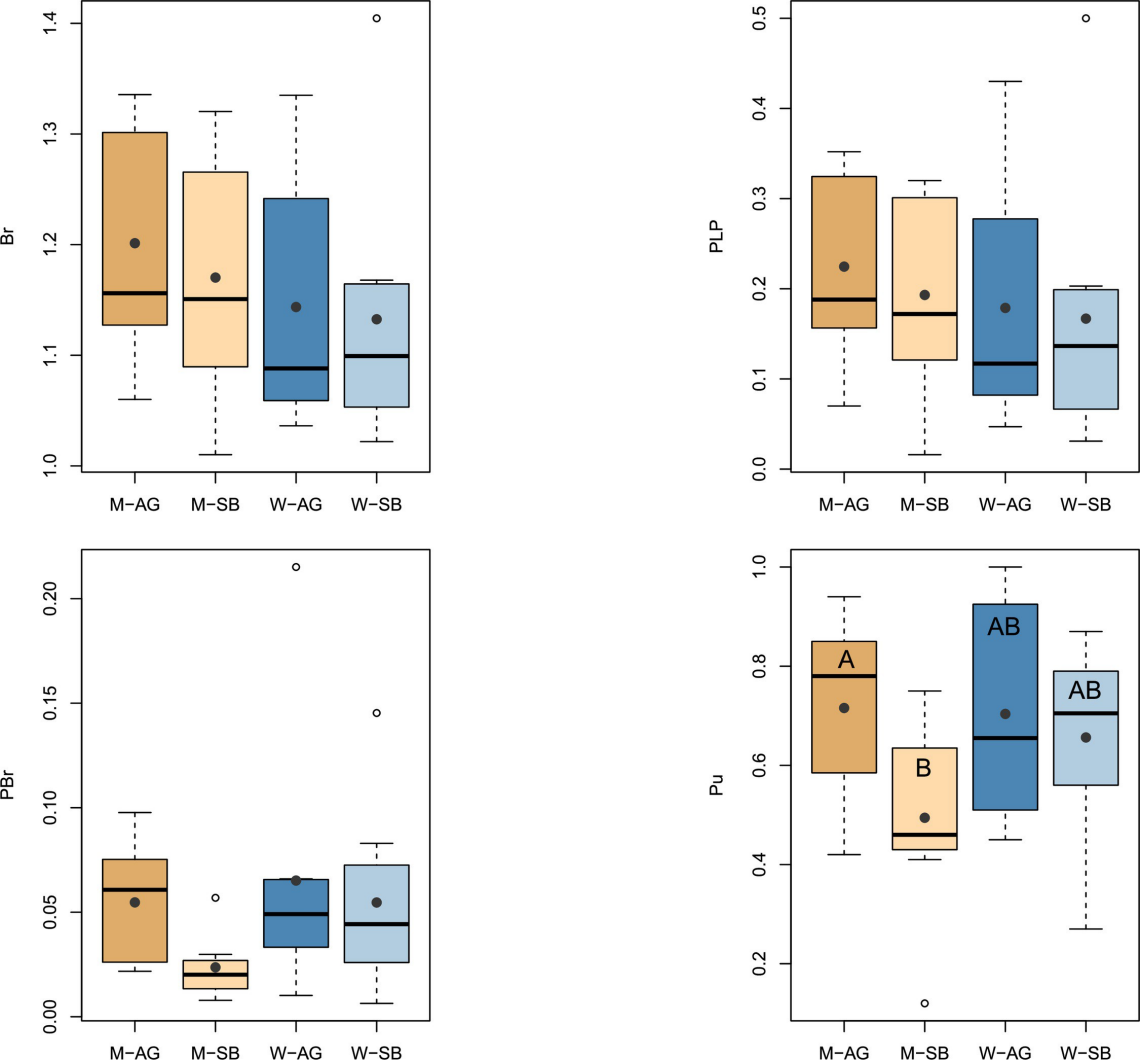
Diversity measures of *Viola elatior* for aboveground and seed bank cohorts in meadow (M) and woodland (W) habitats, respectively. *Br* = band richness, *PLP 5%* = percentage of polymorphic loci at the 5% level; *PBr* = private band richness (aboveground vs. seed bank); *P*_*u*_ = proportion of unique AFLP-phenotypes. Grey diamonds depict arithmetic means.

**Fig 2:**
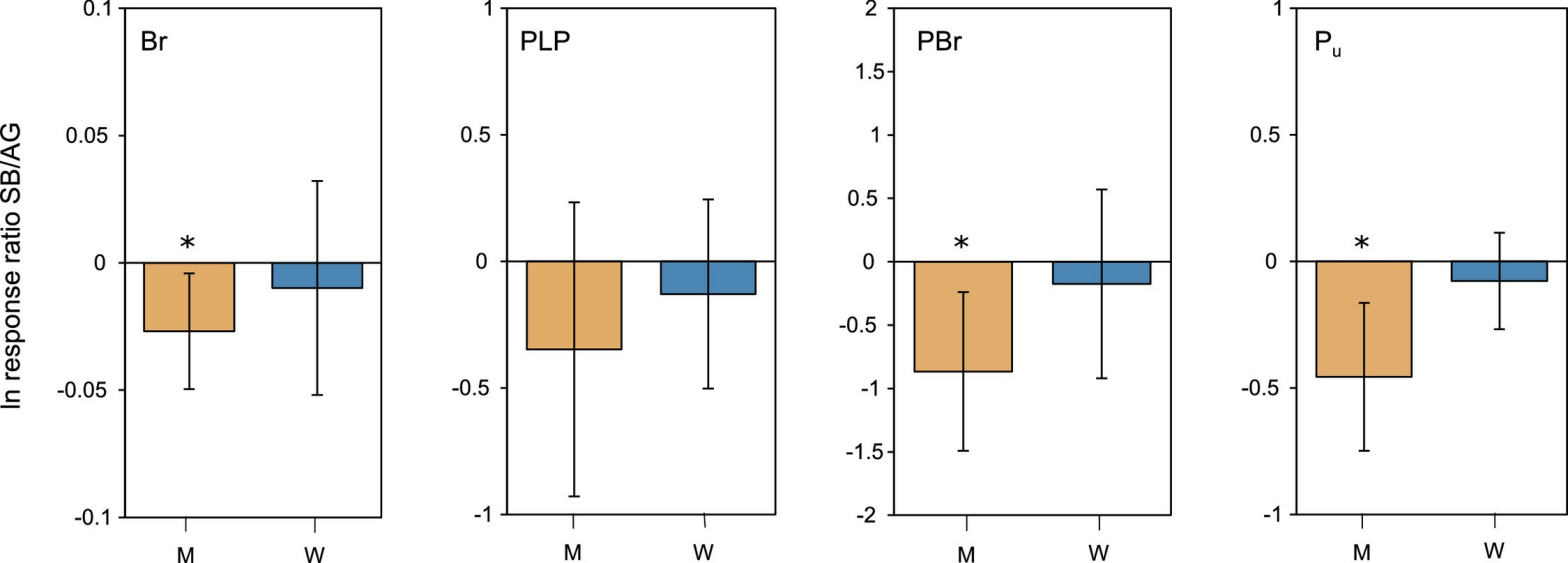
Mean (±95% CI) ln response ratio of *Br*, *PLP*, *PBr*, and *P*_*u*_ between seed bank and aboveground cohorts in meadow (M) and in woodland (W) habitats, respectively. *Br*—band richness, *PLP*—percentage of polymorphic loci at the 5% level; *PBr*—pairwise private band richness (aboveground vs. seed bank); *P*_*u*_—proportion of unique AFLP-phenotypes. Negative ln response ratios denote higher values in the aboveground cohort. Differences between aboveground and seed bank cohorts were considered significant when 95% CI did not overlap with zero.

AFLP analysis resulted in a total of 528 scorable loci of which 128 (24%) were polymorphic which were used for all further calculations. Across the 635 surveyed samples we found 323 unique AFLP phenotypes. After rarefaction, measures of genetic diversity across populations (Table 2) revealed mean values of 1.17 (aboveground) and 1.15 (seed bank) for band richness (*Br*), 20% and 18% for percentage polymorphic loci (*PLP*), 0.06 and 0.04 for private band richness (*PBr*), and 0.71 and 0.58 for proportion of unique genotypes (*P*_*u*_). For *PBr* (LMM, chi^2^ = 4.40, df = 1, p = 0.036) and *P*_*u*_ (LMM, chi^2^ = 15.77, df = 1, p<0.001) the values were significantly higher for aboveground cohorts (Fig 1). Additionally, for *P*_*u*_ there was a significant interaction between habitat and cohort (LMM, chi^2^ = 7.17, df = 1, p = 0.007). The latter was furthermore corroborated by the comparison of log response ratios, indicating for *Br*, *PBr* and *P*_*u*_ significantly higher values for aboveground cohorts in meadow but not in woodland habitats (Fig 2). Individually, three populations had higher genetic diversity values for seed bank than aboveground cohorts for most or all diversity descriptors, notably all in woodland habitats (RW2, TW3, TW5; Table 2).

**Table 2:** Measures of within-population diversity of *Viola elatior* for aboveground and seed bank individuals.

Population	*Br*		*PLP* 5%		*PBr*		*P*_*u*_	
	AG	SB	AG	SB	AG	SB	AG	SB
RM1	1.16	1.12	19	16	0.07	0.03	0.68	0.41
RM2	1.31	1.25	35	31	0.09	0.02	0.86	0.71
RM3	1.34	1.32	34	32	0.03	0.01	0.49	0.45
RM4	1.15	1.15	18	17	0.06	0.06	0.84	0.46
RW1	1.17	1.16	20	20	0.07	0.06	0.92	0.72
RW2	1.06	1.12	9	16	0.03	0.08	0.59	0.79
RW3	1.08	1.06	11	8	0.07	0.04	0.56	0.51
TM1	1.10	1.01	13	2	0.10	0.01	0.42	0.12
TM2	1.06	1.06	7	8	0.02	0.02	0.78	0.56
TM3	1.29	1.28	31	29	0.03	0.02	0.94	0.75
TW1	1.09	1.08	13	11	0.04	0.03	0.93	0.79
TW2	1.05	1.02	8	3	0.04	0.01	0.46	0.27
TW3	1.31	1.40	35	50	0.05	0.15	0.72	0.69
TW4	1.34	1.17	43	20	0.22	0.05	1.00	0.87
TW5	1.04	1.05	5	6	0.01	0.02	0.45	0.61
*Average overall*	*1*.*17*	*1*.*15*	*20*	*18*	*0*.*06*	*0*.*04*	*0*.*71*	*0*.*58*
*Average meadow*	*1*.*20*	*1*.*17*	*22*	*19*	*0*.*06*	*0*.*02*	*0*.*72*	*0*.*49*
*Average woodland*	*1*.*14*	*1*.*13*	*18*	*17*	*0*.*07*	*0*.*05*	*0*.*70*	*0*.*66*
*Average R*	*1*.*18*	*1*.*17*	*21*	*20*	*0*.*06*	*0*.*04*	*0*.*71*	*0*.*58*
*Average T*	*1*.*16*	*1*.*13*	*19*	*16*	*0*.*06*	*0*.*04*	*0*.*71*	*0*.*58*

All variables were calculated after rarefaction to the minimal sample size (12).

AG—aboveground individuals; SB—seed bank individuals; region R—Upper Rhine floodplain; region T—Thaya/Morava floodplain; *Br*—band richness, *PLP 5%*—percentage of polymorphic loci at the 5% level; *PBr*—private band richness (aboveground vs. seed bank); *P*_*u*_—proportion of unique AFLP-phenotypes.

### Genetic structure

Analyses of molecular variance resulted in global ф_ST_ values of 0.80 and 0.83 for aboveground and seed bank cohorts (Table 3), respectively, ranging for population pairwise ф_ST_ between 0.24 and 0.96 for aboveground cohorts and between 0.28 and 0.99 for seed bank cohorts (S3 Table). Hierarchical AMOVA showed that for both cohorts around 16% of genetic variance resided between regions, while most variation (65.8 and 68.7%, respectively) was partitioned among populations within regions (Table 3). Overall, we found very strong genetic differentiation among populations but no significant differentiation between aboveground and seed bank cohorts (S4 Table). Nonetheless, at the individual population level, 2 of 7 meadow populations and 4 of 8 woodland populations exhibited significant genetic differentiation between cohorts with pairwise ф_ST_ values ranging from 0.05 for TW1 to 0.16 for TW4 (S3 Table).

**Table 3:** Summary of hierarchical AMOVA results for aboveground and seed bank cohorts of the surveyed *Viola elatior* populations.

Source	cohort	V	% total	*P*	ф statistics	
Among all populations	AG	14.62	80.06	<0.001		ф_ST_ = 0.80
Among all populations	SB	15.18	83.47	<0.001		ф_ST_ = 0.83
Among regions	AG	3.09	15.69	<0.001	ф_CT_ = 0.16	ф_ST_ = 0.82
Among populations within regions	AG	12.97	65.83	<0.001	ф_SC_ = 0.78	
Within populations	AG	3.64	18.47	<0.001		
Among regions	SB	3.16	16.03	<0.001	ф_CT_ = 0.16	ф_ST_ = 0.85
Among populations within regions	SB	13.53	68.71	<0.001	ф_SC_ = 0.82	
Within populations	SB	3.01	15.27	<0.001		
						

AG—aboveground individuals; SB—seed bank individuals; V—variance components

**Fig 3:**
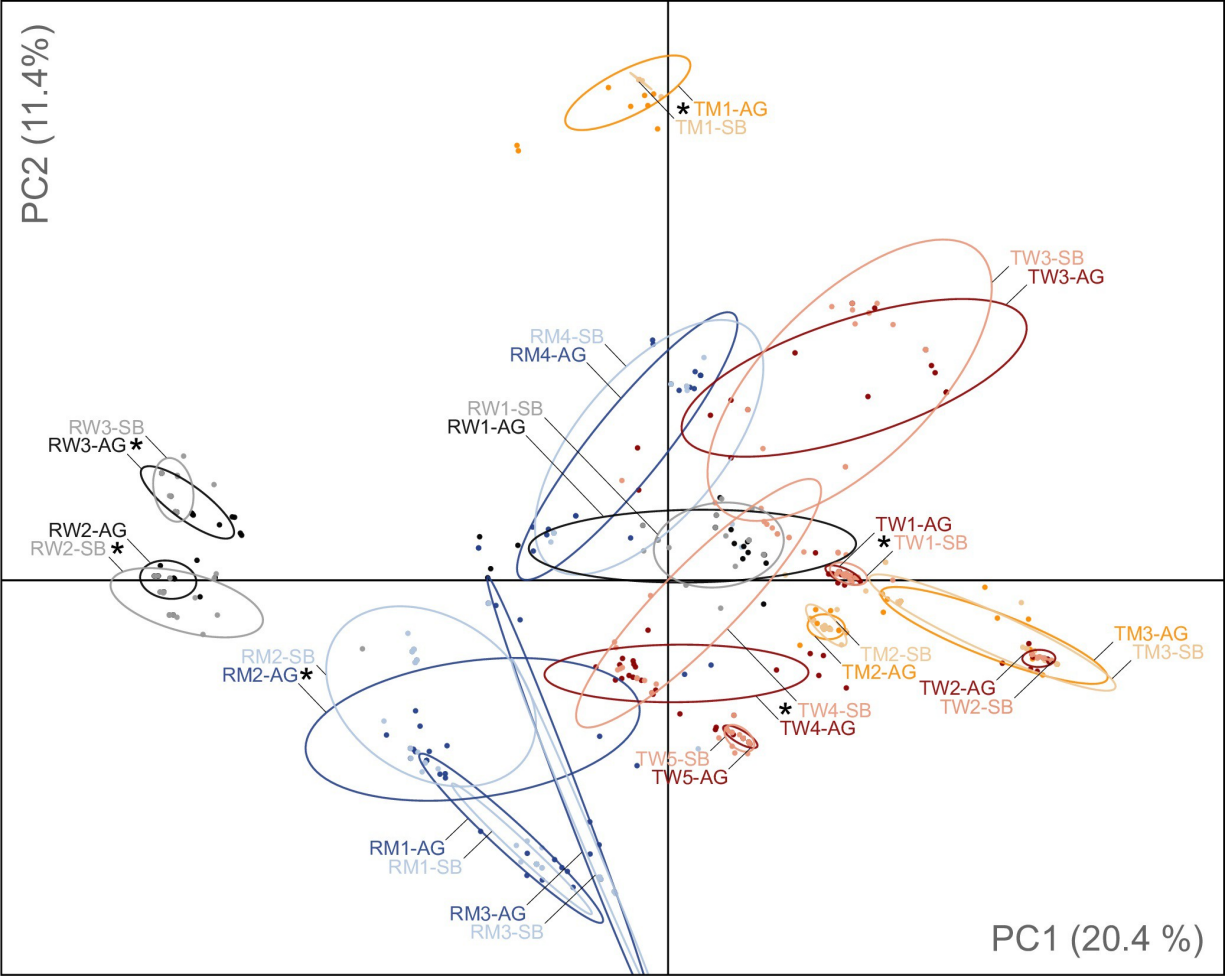
Principal component analysis (PCA) of the genetic structure in populations of *Viola elatior*. German populations are indicated by blue (meadow) and grey (woodland), and Czech populations by orange (meadow) and red (woodland). Aboveground and seed bank cohorts are depicted by strong and light colors, respectively. Inertia ellipses indicate dispersion of samples in relation to mean coordinates and include approximately three-fourths (76%) of all individuals for each group. Stars denote populations with significant genetic differentiation (ф_ST_) between aboveground and seed bank cohorts (see also S3 Table).

Principal component analysis corroborated the AMOVA results, revealing a very close clustering of cohort pairs for most populations (Fig 3). Overall, the first three components accounted for 20.4%, 11.4% and 9.3% of genetic variation. Regions were separated along the first axis, while there was no consistent structuring according to habitat types.

### Small-scale spatial genetic structure

**Fig 4:**
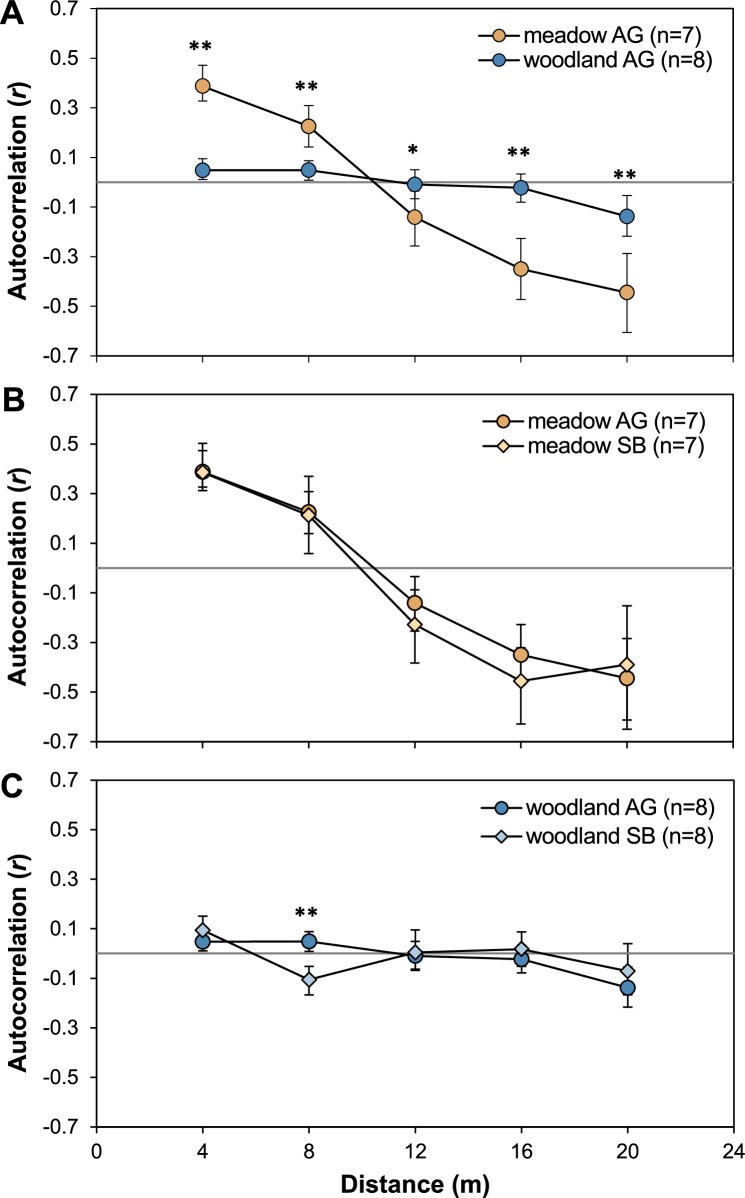
Correlograms of spatial genetic autocorrelation in populations of *Viola elatior*. Comparison of correlogram homogeneity is shown for (A) aboveground populations grouped for habitats, and for aboveground and seed bank populations from either only (B) meadow or (C) woodland habitats; ω-test indicates overall significance (A: ω = 73.74, p = 0.0001; B: ω = 9.33, p = 0.497; C: ω = 19.85, p = 0.030). *p <0.05 and **p<0.01 indicate significant differences for single distance classes. AG—aboveground; SB–seed bank.

For aboveground and seed bank samples each 5 of 15 populations showed significant small-scale spatial genetic structure. *Sp* values ranged from 0.186 to 2.198 for aboveground and from 0.073 to 0.253 for seed bank cohorts (Table 4). Although *Sp* values were lower for seed bank samples, the presence of small-scale spatial genetic structure was comparable among the two cohorts, and in all but one case, populations showing a significant spatial structure at the aboveground level also showed it at the seed bank level. Overall, meadow populations displayed a stronger degree of spatial genetic structure with markedly higher average *Sp* values than woodland populations (aboveground: 0.60 vs. 0.11; seed bank: 0.32 vs. 0.03). This was confirmed by the heterogeneity test with pooled data sets, revealing for both cohorts significantly stronger small-scale spatial genetic structure in meadow than in woodland populations, both overall (Fig 4) and at the regional scale (S1 Fig). Moreover, heterogeneity tests confirmed that small-scale spatial genetic structure was not significantly different between aboveground and seed bank (Fig 4). However, for meadow populations kinship coefficients of aboveground and seed bank samples showed significant differences in the second distance class.

**Table 4:** Small-scale genetic structure for aboveground and seed bank individuals in populations of *Viola elatior*.

Population	n		*F*_(1)_		*b*_log_		*Sp*	
	AG	SB	AG	SB	AG	SB	AG	SB
RM1	21	20	0.000	0.004	-0.004	-0.002	0.004	0.002
RM2	19	7	0.102	0.948	-0.079	-0.085	0.088	1.646
RM3	22	12	0.678**	0.357*	-0.707**	-0.144**	2.198	0.223
RM4	22	18	0.391**	0.425**	-0.428**	-0.062**	0.703	0.108
RW1	23	9	0.329**	0.058	-0.291**	-0.001	0.433	0.001
RW2	20	14	0.016	-0.050	-0.021	0.018	0.021	-0.017
RW3	22	10	0.147	0.358*	-0.159*	-0.085**	0.186	0.133
TM1	21	18	-0.021	-0.019	0.007	0.011	-0.007	-0.011
TM2	21	10	0.041	0.334	-0.134	-0.011	0.140	0.017
TM3	21	20	0.515**	0.548**	-0.487**	-0.114**	1.003	0.253
TW1	23	21	-0.008	-0.059	-0.010	-0.017	0.010	0.016
TW2	23	20	-0.001	-0.109*	-0.042	0.012	0.042	-0.011
TW3	23	19	0.005	0.055	-0.065	-0.013	0.066	0.013
TW4	22	16	0.014	0.265**	-0.008	-0.054*	0.008	0.073
TW5	21	22	0.160*	0.030	-0.067	-0.008	0.079	0.009
*Average*	*21*.*6*	*15*.*7*	*0*.*158*	*0*.*210*	*-0*.*166*	*-0*.*037*	*0*.*332*	*0*.*164*

AG—aboveground individuals; SB—seed bank individuals; n- sample number; *F*_(1)_—kinship coefficient of the first distance class; b_log_—regression slope of spatial genetic autocorrelation; *Sp*—statistic.

Significant values are presented in bold (*p<0.05, **p<0.01).

## Discussion

### Genetic diversity

Genetic diversity of *Viola elatior* was comparably low [48,49]. This is consistent with earlier studies on this species [27,31] and with findings in other plants with predominant seed production through selfed cleistogamous flowers [50,51]. These typically show high levels of inbreeding, little or no genetic variability within populations and strong population differentiation [52].

It is widely assumed that decreasing population sizes lead to a loss of genetic variation through effects of increased random genetic drift, higher inbreeding rates and the accumulation of deleterious mutations (e.g. [53,54]). Furthermore, under changing environmental conditions during succession the loss of genetic variation might be aggravated by an increased probability of local extinction of certain genotypes due to selection [55]. Consequently, we expected to find a decrease of aboveground genetic diversity from meadow to woodland habitats, while seed bank genetic diversity should stay rather constant, given that the seed bank stores genotypes that are lost aboveground. The seed bank reservoir of genotypes might then help to regain former aboveground genetic diversity under more favorable conditions (e.g. after disturbance).

However, we did not find any clear difference of aboveground diversity (Fig 1) between habitats and only cohorts appeared to be significantly different for some of the diversity measures, showing higher aboveground than seed bank values for *PBr* and *P*_*u*_. Overall, these differences can mainly be attributed to changes in meadow habitats. This is substantiated by the significant interaction between habitat and cohort for *P*_*u*_, suggesting that the relationship of the two cohorts changes with ongoing succession. Indeed, using the log-response ratio approach to highlight the relative seed bank genetic diversity (compared to the aboveground cohort) corroborated the results of the linear model for *P*_*u*_. For all diversity measures but PLP, values of meadow populations were significantly higher in aboveground than seed bank cohorts, whereas in woodlands the two cohorts did not differ. Our data thus imply that relative seed bank genetic diversity increases towards the later successional stage.

Generally, higher aboveground than seed bank genetic diversity, as found in meadow populations, might be explained by selection against homozygotes or inbred individuals during germination and recruitment as suggested by earlier seed bank genetic studies [8,16,19]. However, as we did not find reduced seed bank genetic diversity in late successional woodland stages, in which similar or even stronger selection pressure can be expected, we alternatively suggest that in woodland habitats an increase in the relative contribution of outcrossed seeds to the seed bank may be causal for the observed pattern, counteracting effects of selection and reduced population size. Temporal changes in seed bank genetic variation have already been observed for an aerial seed bank [56], presumably due to increased outcrossing rate.

In *V*. *elatior*, several linked processes may have fostered outcrossing in late successional habitats. First, a change in the balance between outcrossed and selfed flowers might have led to higher outcrossing rates in woodland sites. In partially cleistogamous species, the allocation to outcrossing and selfed flowers is controlled by environmental factors [57] and outcrossing often increases with decreasing plant density [58], decreasing light availability [58] and increasing soil water availability [59], i.e. the same environmental differences that are present between meadow and woodland habitats. For the congeneric *Viola mirabilis* increasing outcrossing rates were indeed shown to be correlated with shading [60]. Second, also variation in abortion rates or quality of outcrossed seeds might be important. As in *V*. *elatior* capsules from outcrossed flowers mature only in the warmest period of the year, differences in water availability between meadow and woodland habitats are particularly distinct during the development of outcrossed seeds. This is corroborated by a survey in four populations from the Upper Rhine region (Schulz, unpublished data) indicating for seeds from outcrossing flowers higher abortion rates (30% vs. 6%) as well as lower seed mass (10 mg vs. 18 mg) in meadow than in woodland sites. In contrast, in the same populations differences for seeds from selfed flowers were less pronounced (seed abortion: 4.8% vs. 2.2%; seed mass: 15 mg vs. 17 mg). Additionally, seed longevity might be higher in woodland habitats, as soil parameters like high moisture content and constant temperatures are beneficial for seed survival. Thus, woodland seed banks potentially could be assembled from more seed generations, leading to higher relative genetic diversity. Finally, also anthropogenic effects may have an impact on the relative contribution of seeds from outcrossing flowers. As most of the floodplain meadows are regularly managed through mowing in early June, large amounts of ripening capsules from outcrossing flowers will be destroyed every year, whereas capsules from selfed flowers can freely develop from July to October, likely leading to a lower proportion of outcrossed seeds in meadow compared to woodland seed banks.

In accordance with Eckstein et al. [31], we found no significant differences in genetic diversity between German and Czech populations, neither for aboveground nor seed bank cohorts. This strongly implies that the detected habitat related differences seem to present a general effect that is independent of geographic location.

### Genetic structure

Genetic differentiation in *Viola elatior* was very high, with 80.1% and 83.5% of genetic variation residing among populations for aboveground and seed bank cohorts, respectively. Similar differentiation (up to 82%) has been also reported in earlier studies on this species [27,31,61], reflecting the predominant selfing breeding system and relatively small population sizes and hence a strong influence of genetic drift on population structure. This is further substantiated by a lack of correlation between genetic differentiation and geographic distances in populations from the Upper Rhine Valley [27], which indicates that gene flow is much too low to counteract population divergence by genetic drift. Overall, population differentiation was virtually identical among seed bank and aboveground cohorts at all hierarchical levels (Table 3). In contrast, some other studies reported lower differentiation among seed bank cohorts than among aboveground cohorts [6,9,62], indicating that aboveground populations can become more differentiated than the homogeneous seed pools they derived from [9]. Accordingly, in *V*. *elatior* habitat related change in selection was not as high as expected and differentiation between cohorts is an exception that occurred only in few individual populations.

### Small-scale spatial genetic structure

Spatial genetic structure within populations arises due to spatially restricted gene dispersal and is mainly related to the amount of gene flow by seeds and pollen [63]. Therefore, the observed significant decrease of small-scale spatial genetic structure from meadow to woodland habitats strongly supports the assumption that in *Viola elatior* the relative impact of outcrossing and thus pollen dispersal increases towards late successional stages. High selfing rates in meadow populations are furthermore corroborated by high *Sp* values that even exceeded mean values reported for other predominantly selfing species (*Sp* = 0.14, [44]). In contrast, *Sp* values in woodland populations were markedly lower and do match better with values for mixed mating species (*Sp* = 0.04, [44]). Thus, if in meadow populations almost all seeds result from self-fertilization, only seed dispersal (average distance ~1.3 m, [26]) does impact gene dispersal distances. In contrast, in woodland populations and assuming considerable outcrossing, both seed and pollen dispersal contribute to, and increase, gene dispersal [44]. Both, aboveground and seed bank cohorts showed significant small-scale spatial genetic structure and even though *Sp* values were generally lower in seed bank cohorts, overall there were no significant differences between the two groups. Similar to our results, other studies also detected significant small-scale spatial genetic structure in both cohorts [15,17,64] suggesting that the spatial structure of aboveground and seed bank individuals is often mutually dependent ([17], but see [16]).

Taken together, the observed small-scale spatial genetic structure in *Viola elatior* likely reflects the proposed differences in outcrossing rates between habitats. However, generally spatial genetic structure appears to break down relatively fast with ongoing succession and is not stored over longer periods of time in seed bank cohorts. Otherwise much stronger spatial autocorrelation would have been present in the seed bank of woodland populations. Instead, the high correlation of aboveground and seed bank cohorts in both habitats suggests that turnover of seeds in soil and of adult plants is similar in *V*. *elatior* and that seeds do not persist in the seed bank for more than few adult generations [6].

## Conclusions

To our knowledge, the present study is the first one that has compared aboveground and seed bank genetic diversity between successional stages. Surveying a relative large number of populations of the partially cleistogamous *Viola elatior* in two different regions, we could show that the contribution of outcrossing to reproduction seems to increase from early to late successional stages, leading to higher relative seed bank diversity and lower within population small-scale spatial genetic structure. This suggests that under favorable early successional conditions with high plant densities, populations maintain their approved genotypes mainly by selfing. Under less favorable conditions an increase in outcrossing may keep genetic diversity in *V*. *elatior* at a constant level and hence seems to compensate the detrimental effects of small population size. Ultimately, in late successional habitats with a higher risk of extinction, the resulting increased relative seed bank genetic diversity also assures a higher chance for population recovery and thus the long-term persistence of the species.

We suggest that the relationship between seed bank and above ground plants potentially can be driven by both, post-germination selection as seen in meadow habitats and genetic buffering through stored seeds that seems to counteract the effects of drift and selection as seen in woodland populations. However, to substantiate these results and to test if this is not a specific situation in partially cleistogamous plants, further studies surveying seed bank genetics along environmental gradients in species with other mating systems are needed.

## Supporting information

S1 Text AFLP protocol. (DOCX)

S1 Table Adaptor- and primer sequences used for AFLP analyses. (DOCX)

S2 Table Seedling emergence rates of *Viola elatior* in soil samples from the surveyed populations. (DOCX)

S3 Table Pairwise ф_ST_-matrix for aboveground and seed bank cohorts of the surveyed populations. (DOCX)

S4 Table Summary of hierarchical AMOVA results for German and Czech populations grouped by cohort type. (DOCX)

S1 Fig Correlograms of genetic autocorrelation in populations of *Viola elatior*. Comparison of correlogram homogeneity is shown for (A) Czech and (B) German aboveground populations grouped for habitats, respectively; ω-test indicates overall significance (A: ω = 67.18, p = 0.0001; B: ω = 33.91, p = 0.0003). *p<0.05 and **p<0.01 indicate significant differences for single distance classes. Cz—Czech Republic; Ger–Germany; AG—aboveground.
(TIF)
