# Supplementary material for: Differential role of a persistent seed bank for genetic variation in early vs. late successional stages
Source: PLoS One. 2018 Dec 26;13(12):e0209840. doi: 10.1371/journal.pone.0209840 (PMC6306206; doi:10.1371/journal.pone.0209840)
Supplement: S1 Text — (DOCX) [file pone.0209840.s001.docx]

**S1 Text.** AFLP protocol.

For restriction and ligation (RL) 5.6 μl genomic DNA were combined with 5.4 μl RL reaction mix containing 0.55 μl BSA (1 mg/ml; New England Biolabs, NEB), 1.1 μl 0.5 M NaCl, 5 u *Eco*RI (NEB), 1 u *Mse*I (NEB), 67 u T4 DNA ligase (NEB), 1.1 μl T4 DNA ligase buffer (NEB), 1 μl *Eco*RI adapter (5 pmol) and 1 μl *Mse*I adapter (50 pmol). The reaction was incubated for 2 h at 37 °C and diluted 1:2. For the preselective amplification (PCR1), 4 μl RL product were combined with 16 μl PCR1 reaction mix containing 1.5 ng/μl *Eco*RI- and *Mse*I preselective primers each, 200 μM dNTPs (Roth), 2 μl 10 x Dream Tag buffer (QIAGEN), 0.8 u Dream Tag polymerase (QIAGEN) and 9.84 μl H_2_O. The thermocycler protocol was 72.0°C (2 min) followed by 20 cycles of 94.0°C (20 s), 56.0°C (30 s) and 72.0°C (2 min) and a final extension at 60.0°C (30 min), performed on an Eppendorf Mastercycler gradient. The PCR1 product was diluted 1:5. For the selective amplification (PCR2), 1 μl PCR1 product was combined with 3.4 μl PCR2 reaction mix containing 2.2 μl Multiplex PCR kit (QIAGEN) and 0.6 μl fluorescent labeled *Eco*RI primer (1 pmol/μl) and 0.6 μl *Mse*I (5 pmol/μl) selective primers each. The thermocycler protocol was 94.0°C (2 min) followed by 10 cycles of 94.0°C (20 s), 66.0°C (30 s, decreasing 1°C per cycle) and 72.0°C (2 min) and 20 cycles of 94.0°C (20 s), 56.0°C (30 s) and 72.0°C (2 min), and a final extension at 60.0°C (30 min), performed on an Eppendorf Mastercycler pro 384.
