# Supplementary material for: Differential role of a persistent seed bank for genetic variation in early vs. late successional stages
Source: PLoS One. 2018 Dec 26;13(12):e0209840. doi: 10.1371/journal.pone.0209840 (PMC6306206; doi:10.1371/journal.pone.0209840)
Supplement: S1 Table — (DOCX) [file pone.0209840.s002.docx]

**S1 Table.** Adaptor- and primer sequences used for AFLP analyses.

| **Primer** |  |  | |  | **Sequence** |  |  |  |
| --- | --- | --- | --- | --- | --- | --- | --- | --- |
|  |  |  | |  |  |  |  |  |
| Adaptors |  |  | |  |  |  |  |  |
|  | *EcoR*I*-adapter* top | | |  | 5'-CTCGTAGACTGCGTACC-3' | | | |
|  | *EcoR*I*-adapter* bottom | | | | 5'-AATTGGTACGCAGTCTAC-3' | | | |
|  | *Mse*I*-adapter* top | | | | 5'-GAGCGATGAGTCCTGAG-3' | | | |
|  | *Mse*I*-adapter* bottom | | | | 3'-TACTCAGGACTCAT-5' | | |  |
|  |  | |  |  |  |  |  |  |
| Preselective primers | | |  |  |  |  |  |  |
|  | *Eco*RI + A | | |  | 5'-GACTGCGTACCAATTCA-3' | | | |
|  | *Mse*I + C | | |  | 5'-GATGAGTCCTGAGTAAC-3' | | | |
|  |  |  | |  |  |  |  |  |
| Selective primer | | | |  |  |  |  |  |
|  | *Eco*RI + AAC-FAM*^1^ | | | | 5'-GACTGCGTACCAATTCAAC-3' | | | |
|  | *Eco*RI + ACT-FAM^2^ | | | | 5'-GACTGCGTACCAATTCACT-3' | | | |
|  | *Eco*RI + ACA-VIC^3,4^ | | | | 5'-GACTGCGTACCAATTCACA-3' | | | |
|  | *Eco*RI + AAG-NED^5,6^ | | | | 5'-GACTGCGTACCAATTCAAG-3' | | | |
|  | *Eco*RI + AGC-PET^7^ | | | | 5'-GACTGCGTACCAATTCAGC-3' | | | |
|  | *Eco*RI + AGG-PET^8^ | | | | 5'-GACTGCGTACCAATTCAGG-3' | | | |
|  | *Mse*I + CTA^1^ | | |  | 5'-GATGAGTCCTGAGTAACTA-3' | | | |
|  | *Mse*I + CAA^2,3,7^ | | |  | 5'-GATGAGTCCTGAGTAACAA-3' | | | |
|  | *Mse*I + CAC^5^ | | |  | 5'-GATGAGTCCTGAGTAACAC-3' | | | |
|  | *Mse*I + CTC^4,6,8^ | | |  | 5'-GATGAGTCCTGAGTAACTC-3' | | | |
|  |  |  | |  |  |  |  |  |
|  |  | | | |  | | | |

*Superscript numbers indicate primer combinations used for the selective amplification
