# Supplementary material for: Differential role of a persistent seed bank for genetic variation in early vs. late successional stages
Source: PLoS One. 2018 Dec 26;13(12):e0209840. doi: 10.1371/journal.pone.0209840 (PMC6306206; doi:10.1371/journal.pone.0209840)
Supplement: S2 Table — (DOCX) [file pone.0209840.s003.docx]

**S2 Table.** Seedling emergence rates of *Viola elatior* in soil samples from the surveyed populations.

| Population | Total seedlings | Seedlings per m^2^ |
| --- | --- | --- |
| RM1 | 20 | 66.7 |
| RM2 | 26 | 86.7 |
| RM3 | 12 | 40.0 |
| RM4 | 18 | 60.0 |
| RW1 | 26 | 86.7 |
| RW2 | 44 | 146.7 |
| RW3 | 28 | 93.3 |
| TM1 | 61 | 203.3 |
| TM2 | 116 | 386.7 |
| TM3 | 63 | 210.0 |
| TW1 | 156 | 520.0 |
| TW2 | 206 | 686.7 |
| TW3 | 72 | 240.0 |
| TW4 | 85 | 283.3 |
| TW5 | 79 | 263.3 |
