# Supplementary material for: Differential role of a persistent seed bank for genetic variation in early vs. late successional stages
Source: PLoS One. 2018 Dec 26;13(12):e0209840. doi: 10.1371/journal.pone.0209840 (PMC6306206; doi:10.1371/journal.pone.0209840)
Supplement: S3 Table — (DOCX) [file pone.0209840.s004.docx]

**S3 Table.** Pairwise ф_ST_-matrix for aboveground and seed bank cohorts of the surveyed populations.

|  | RM1-AG | RM1-SB | RM2-AG | RM2-SB | RM3-AG | RM3-SB | RM4-AG | RM4-SB | RW1-AG | RW1-SB | RW2-AG | RW2-SB | RW3-AG | RW3-SB | TM1-AG | TM1-SB | TM2-AG | TM2-SB | TM3-AG | TM3-SB | TW1-AG | TW1-SB | TW2-AG | TW2-SB | TW3-AG | TW3-SB | TW4-AG | TW4-SB | TW5-AG | TW5-SB |
| --- | --- | --- | --- | --- | --- | --- | --- | --- | --- | --- | --- | --- | --- | --- | --- | --- | --- | --- | --- | --- | --- | --- | --- | --- | --- | --- | --- | --- | --- | --- |
| RM1-AG | - | **0.17** | 0.00 | 0.00 | 0.00 | 0.00 | 0.00 | 0.00 | 0.00 | 0.00 | 0.00 | 0.00 | 0.00 | 0.00 | 0.00 | 0.00 | 0.00 | 0.00 | 0.00 | 0.00 | 0.00 | 0.00 | 0.00 | 0.00 | 0.00 | 0.00 | 0.00 | 0.00 | 0.00 | 0.00 |
| RM1-SB | **0.03** | - | 0.00 | 0.00 | 0.00 | 0.00 | 0.00 | 0.00 | 0.00 | 0.00 | 0.00 | 0.00 | 0.00 | 0.00 | 0.00 | 0.00 | 0.00 | 0.00 | 0.00 | 0.00 | 0.00 | 0.00 | 0.00 | 0.00 | 0.00 | 0.00 | 0.00 | 0.00 | 0.00 | 0.00 |
| RM2-AG | 0.62 | 0.67 | - | **0.03** | 0.00 | 0.00 | 0.00 | 0.00 | 0.00 | 0.00 | 0.00 | 0.00 | 0.00 | 0.00 | 0.00 | 0.00 | 0.00 | 0.00 | 0.00 | 0.00 | 0.00 | 0.00 | 0.00 | 0.00 | 0.00 | 0.00 | 0.00 | 0.00 | 0.00 | 0.00 |
| RM2-SB | 0.67 | 0.71 | **0.11** | - | 0.00 | 0.00 | 0.00 | 0.00 | 0.00 | 0.00 | 0.00 | 0.00 | 0.00 | 0.00 | 0.00 | 0.00 | 0.00 | 0.00 | 0.00 | 0.00 | 0.00 | 0.00 | 0.00 | 0.00 | 0.00 | 0.00 | 0.00 | 0.00 | 0.00 | 0.00 |
| RM3-AG | 0.53 | 0.56 | 0.51 | 0.56 | - | **0.43** | 0.00 | 0.00 | 0.00 | 0.00 | 0.00 | 0.00 | 0.00 | 0.00 | 0.00 | 0.00 | 0.00 | 0.00 | 0.00 | 0.00 | 0.00 | 0.00 | 0.00 | 0.00 | 0.00 | 0.00 | 0.00 | 0.00 | 0.00 | 0.00 |
| RM3-SB | 0.63 | 0.69 | 0.58 | 0.64 | **-0.03** | - | 0.00 | 0.00 | 0.00 | 0.00 | 0.00 | 0.00 | 0.00 | 0.00 | 0.00 | 0.00 | 0.00 | 0.00 | 0.00 | 0.00 | 0.00 | 0.00 | 0.00 | 0.00 | 0.00 | 0.00 | 0.00 | 0.00 | 0.00 | 0.00 |
| RM4-AG | 0.82 | 0.85 | 0.68 | 0.70 | 0.70 | 0.77 | - | **0.32** | 0.00 | 0.00 | 0.00 | 0.00 | 0.00 | 0.00 | 0.00 | 0.00 | 0.00 | 0.00 | 0.00 | 0.00 | 0.00 | 0.00 | 0.00 | 0.00 | 0.00 | 0.00 | 0.00 | 0.00 | 0.00 | 0.00 |
| RM4-SB | 0.83 | 0.87 | 0.69 | 0.72 | 0.70 | 0.78 | **0.00** | - | 0.00 | 0.00 | 0.00 | 0.00 | 0.00 | 0.00 | 0.00 | 0.00 | 0.00 | 0.00 | 0.00 | 0.00 | 0.00 | 0.00 | 0.00 | 0.00 | 0.00 | 0.00 | 0.00 | 0.00 | 0.00 | 0.00 |
| RW1-AG | 0.79 | 0.83 | 0.65 | 0.69 | 0.61 | 0.72 | 0.75 | 0.76 | - | **0.23** | 0.00 | 0.00 | 0.00 | 0.00 | 0.00 | 0.00 | 0.00 | 0.00 | 0.00 | 0.00 | 0.00 | 0.00 | 0.00 | 0.00 | 0.00 | 0.00 | 0.00 | 0.00 | 0.00 | 0.00 |
| RW1-SB | 0.81 | 0.85 | 0.68 | 0.71 | 0.62 | 0.73 | 0.77 | 0.78 | **0.01** | - | 0.00 | 0.00 | 0.00 | 0.00 | 0.00 | 0.00 | 0.00 | 0.00 | 0.00 | 0.00 | 0.00 | 0.00 | 0.00 | 0.00 | 0.00 | 0.00 | 0.00 | 0.00 | 0.00 | 0.00 |
| RW2-AG | 0.88 | 0.92 | 0.74 | 0.72 | 0.74 | 0.85 | 0.87 | 0.89 | 0.88 | 0.89 | - | **0.00** | 0.00 | 0.00 | 0.00 | 0.00 | 0.00 | 0.00 | 0.00 | 0.00 | 0.00 | 0.00 | 0.00 | 0.00 | 0.00 | 0.00 | 0.00 | 0.00 | 0.00 | 0.00 |
| RW2-SB | 0.85 | 0.90 | 0.70 | 0.69 | 0.72 | 0.82 | 0.85 | 0.87 | 0.85 | 0.87 | **0.16** | - | 0.00 | 0.00 | 0.00 | 0.00 | 0.00 | 0.00 | 0.00 | 0.00 | 0.00 | 0.00 | 0.00 | 0.00 | 0.00 | 0.00 | 0.00 | 0.00 | 0.00 | 0.00 |
| RW3-AG | 0.86 | 0.91 | 0.74 | 0.74 | 0.73 | 0.83 | 0.85 | 0.87 | 0.85 | 0.87 | 0.71 | 0.64 | - | **0.01** | 0.00 | 0.00 | 0.00 | 0.00 | 0.00 | 0.00 | 0.00 | 0.00 | 0.00 | 0.00 | 0.00 | 0.00 | 0.00 | 0.00 | 0.00 | 0.00 |
| RW3-SB | 0.88 | 0.92 | 0.75 | 0.75 | 0.74 | 0.85 | 0.86 | 0.88 | 0.87 | 0.88 | 0.74 | 0.67 | **0.09** | - | 0.00 | 0.00 | 0.00 | 0.00 | 0.00 | 0.00 | 0.00 | 0.00 | 0.00 | 0.00 | 0.00 | 0.00 | 0.00 | 0.00 | 0.00 | 0.00 |
| TM1-AG | 0.90 | 0.93 | 0.81 | 0.82 | 0.78 | 0.87 | 0.86 | 0.87 | 0.86 | 0.87 | 0.94 | 0.92 | 0.92 | 0.93 | - | **0.00** | 0.00 | 0.00 | 0.00 | 0.00 | 0.00 | 0.00 | 0.00 | 0.00 | 0.00 | 0.00 | 0.00 | 0.00 | 0.00 | 0.00 |
| TM1-SB | 0.93 | 0.96 | 0.85 | 0.86 | 0.82 | 0.91 | 0.89 | 0.91 | 0.90 | 0.91 | 0.97 | 0.95 | 0.96 | 0.97 | **0.06** | - | 0.00 | 0.00 | 0.00 | 0.00 | 0.00 | 0.00 | 0.00 | 0.00 | 0.00 | 0.00 | 0.00 | 0.00 | 0.00 | 0.00 |
| TM2-AG | 0.89 | 0.93 | 0.82 | 0.83 | 0.77 | 0.86 | 0.86 | 0.87 | 0.87 | 0.88 | 0.95 | 0.92 | 0.93 | 0.95 | 0.94 | 0.97 | - | **0.75** | 0.00 | 0.00 | 0.00 | 0.00 | 0.00 | 0.00 | 0.00 | 0.00 | 0.00 | 0.00 | 0.00 | 0.00 |
| TM2-SB | 0.90 | 0.94 | 0.83 | 0.84 | 0.79 | 0.87 | 0.87 | 0.88 | 0.88 | 0.89 | 0.95 | 0.93 | 0.94 | 0.95 | 0.95 | 0.98 | **-0.02** | - | 0.00 | 0.00 | 0.00 | 0.00 | 0.00 | 0.00 | 0.00 | 0.00 | 0.00 | 0.00 | 0.00 | 0.00 |
| TM3-AG | 0.73 | 0.76 | 0.63 | 0.66 | 0.60 | 0.65 | 0.70 | 0.70 | 0.64 | 0.65 | 0.83 | 0.81 | 0.83 | 0.83 | 0.79 | 0.83 | 0.72 | 0.74 | - | **0.81** | 0.00 | 0.00 | 0.00 | 0.00 | 0.00 | 0.00 | 0.00 | 0.00 | 0.00 | 0.00 |
| TM3-SB | 0.73 | 0.75 | 0.62 | 0.65 | 0.60 | 0.65 | 0.69 | 0.69 | 0.63 | 0.63 | 0.83 | 0.80 | 0.82 | 0.82 | 0.78 | 0.82 | 0.71 | 0.73 | **-0.04** | - | 0.00 | 0.00 | 0.00 | 0.00 | 0.00 | 0.00 | 0.00 | 0.00 | 0.00 | 0.00 |
| TW1-AG | 0.88 | 0.92 | 0.80 | 0.83 | 0.77 | 0.85 | 0.86 | 0.87 | 0.86 | 0.87 | 0.95 | 0.93 | 0.93 | 0.94 | 0.93 | 0.96 | 0.93 | 0.93 | 0.75 | 0.74 | - | **0.03** | 0.00 | 0.00 | 0.00 | 0.00 | 0.00 | 0.00 | 0.00 | 0.00 |
| TW1-SB | 0.89 | 0.92 | 0.80 | 0.83 | 0.77 | 0.86 | 0.86 | 0.88 | 0.86 | 0.88 | 0.95 | 0.93 | 0.93 | 0.94 | 0.93 | 0.96 | 0.93 | 0.94 | 0.75 | 0.73 | **0.05** | - | 0.00 | 0.00 | 0.00 | 0.00 | 0.00 | 0.00 | 0.00 | 0.00 |
| TW2-AG | 0.90 | 0.94 | 0.81 | 0.83 | 0.75 | 0.85 | 0.87 | 0.89 | 0.84 | 0.86 | 0.96 | 0.95 | 0.95 | 0.96 | 0.95 | 0.98 | 0.93 | 0.94 | 0.24 | 0.26 | 0.93 | 0.93 | - | **0.67** | 0.00 | 0.00 | 0.00 | 0.00 | 0.00 | 0.00 |
| TW2-SB | 0.91 | 0.95 | 0.82 | 0.84 | 0.76 | 0.87 | 0.88 | 0.90 | 0.86 | 0.87 | 0.97 | 0.95 | 0.96 | 0.97 | 0.96 | 0.99 | 0.95 | 0.95 | 0.25 | 0.28 | 0.94 | 0.94 | **-0.01** | - | 0.00 | 0.00 | 0.00 | 0.00 | 0.00 | 0.00 |
| TW3-AG | 0.75 | 0.78 | 0.68 | 0.70 | 0.62 | 0.68 | 0.69 | 0.69 | 0.66 | 0.66 | 0.83 | 0.81 | 0.80 | 0.81 | 0.73 | 0.77 | 0.76 | 0.77 | 0.50 | 0.49 | 0.77 | 0.77 | 0.70 | 0.71 | - | **0.27** | 0.00 | 0.00 | 0.00 | 0.00 |
| TW3-SB | 0.73 | 0.76 | 0.66 | 0.67 | 0.60 | 0.66 | 0.67 | 0.67 | 0.63 | 0.63 | 0.80 | 0.79 | 0.78 | 0.78 | 0.70 | 0.74 | 0.72 | 0.73 | 0.46 | 0.46 | 0.73 | 0.72 | 0.67 | 0.68 | **0.01** | - | 0.00 | 0.00 | 0.00 | 0.00 |
| TW4-AG | 0.73 | 0.76 | 0.58 | 0.60 | 0.61 | 0.67 | 0.69 | 0.70 | 0.72 | 0.73 | 0.80 | 0.77 | 0.80 | 0.80 | 0.82 | 0.85 | 0.78 | 0.79 | 0.59 | 0.58 | 0.77 | 0.77 | 0.77 | 0.78 | 0.64 | 0.62 | - | **0.00** | 0.00 | 0.00 |
| TW4-SB | 0.81 | 0.85 | 0.67 | 0.69 | 0.69 | 0.76 | 0.75 | 0.76 | 0.78 | 0.80 | 0.87 | 0.84 | 0.86 | 0.87 | 0.86 | 0.89 | 0.84 | 0.85 | 0.66 | 0.65 | 0.85 | 0.62 | 0.84 | 0.86 | 0.71 | 0.68 | **0.16** | - | 0.00 | 0.00 |
| TW5-AG | 0.89 | 0.93 | 0.78 | 0.80 | 0.74 | 0.84 | 0.89 | 0.90 | 0.86 | 0.88 | 0.95 | 0.93 | 0.94 | 0.95 | 0.95 | 0.98 | 0.94 | 0.94 | 0.72 | 0.71 | 0.94 | 0.94 | 0.95 | 0.96 | 0.78 | 0.75 | 0.77 | 0.85 | - | **0.79** |
| TW5-SB | 0.88 | 0.92 | 0.78 | 0.80 | 0.74 | 0.84 | 0.88 | 0.90 | 0.86 | 0.87 | 0.95 | 0.92 | 0.93 | 0.95 | 0.94 | 0.97 | 0.93 | 0.94 | 0.72 | 0.71 | 0.93 | 0.93 | 0.94 | 0.95 | 0.78 | 0.75 | 0.77 | 0.84 | **-0.02** | - |
| Lower and upper diagonal represent pairwise ф_ST_ and corresponding *p* values after 9999 permutations, respectively; light and dark gray cells depict non-significant and significant ф_ST_ *p* values for population pairs of aboveground (AG) and seed bank (SB) individuals, respectively. | | | | | | | | | | | | | | | | | | | | | | | | | | | | | | |
