# Supplementary material for: Differential role of a persistent seed bank for genetic variation in early vs. late successional stages
Source: PLoS One. 2018 Dec 26;13(12):e0209840. doi: 10.1371/journal.pone.0209840 (PMC6306206; doi:10.1371/journal.pone.0209840)
Supplement: S4 Table — (DOCX) [file pone.0209840.s005.docx]

**S4 Table.** Summary of hierarchical AMOVA results for German and Czech populations grouped by cohort type.

| **Source** | **region** | **V** | **% total** | ***P*** | **ф statistics** |
| --- | --- | --- | --- | --- | --- |
|  |  |  |  |  |  |
| Among cohorts | Ger | -1.80 | -12.43 | 0.994 | ф_CT_ = -0.12 |
| Among populations within cohorts | Ger | 12.60 | 87.12 | <0.001 | ф_SC_ = 0.77 |
| Within populations | Ger | 3.66 | 25.30 | <0.001 | ф_ST_ = 0.75 |
|  |  |  |  |  |  |
| Among cohorts | Cz | -1.70 | -11.22 | 0.982 | ф_CT_ = -0.11 |
| Among populations within cohorts | Cz | 13.74 | 90.9 | <0.001 | ф_SC_ = 0.82 |
| Within populations | Cz | 3.07 | 20.32 | <0.001 | ф_ST_ = 0.80 |
|  |  |  |  |  |  |
| Ger - Germany; Cz - Czech Republic; V – variance components | | | | | |
