# Supplementary figures and images for: Differential role of a persistent seed bank for genetic variation in early vs. late successional stages
Source: PLoS One. 2018 Dec 26;13(12):e0209840. doi: 10.1371/journal.pone.0209840 (PMC6306206; doi:10.1371/journal.pone.0209840)

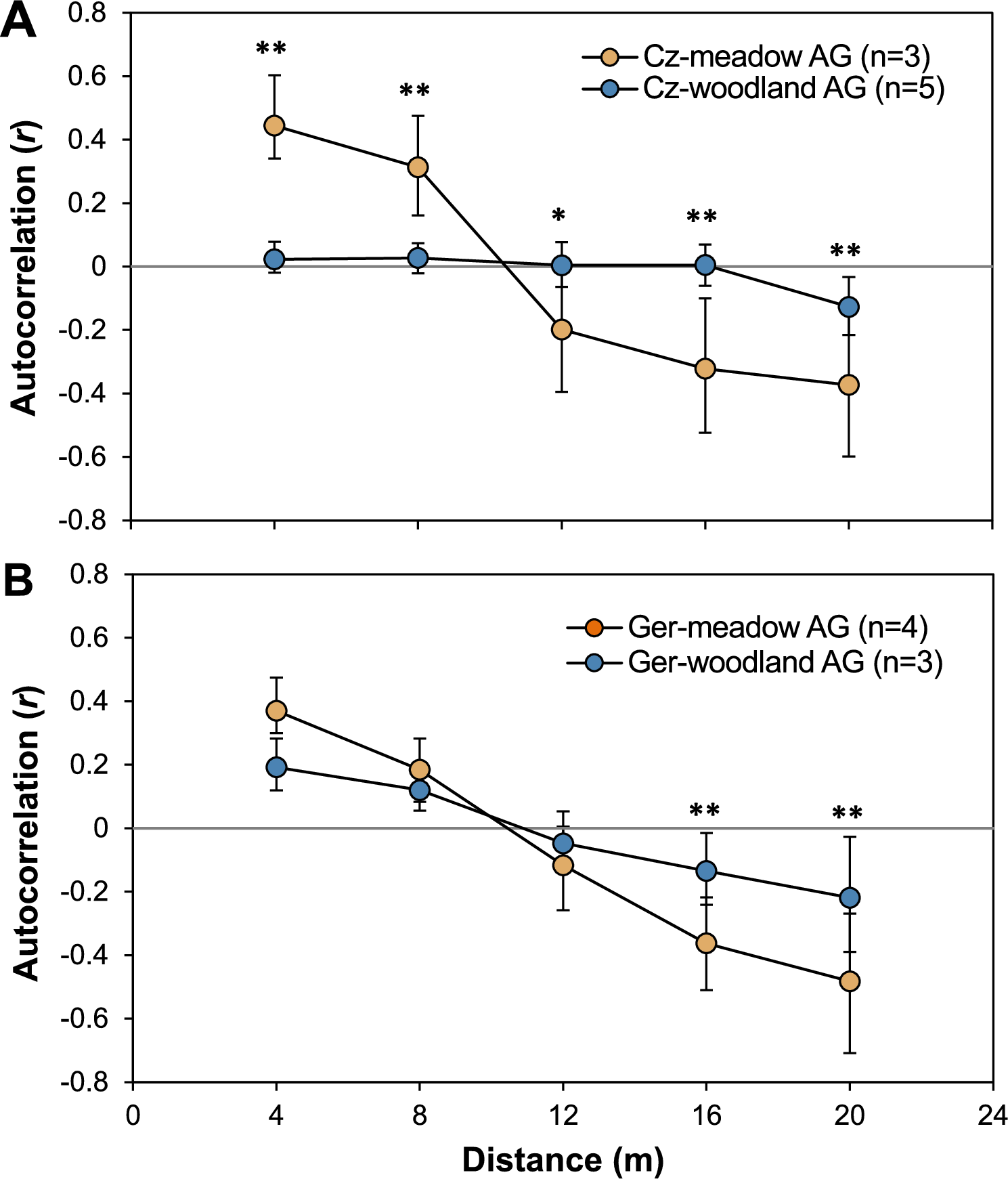

Supplement: S1 Fig — Comparison of correlogram homogeneity is shown for (A) Czech and (B) German aboveground populations grouped for habitats, respectively; ω-test indicates overall significance (A: ω = 67.18, p = 0.0001; B: ω = 33.91, p = 0.0003). *p<0.05 and **p<0.01 indicate significant differences for single distance classes. Cz—Czech Republic; Ger–Germany; AG—aboveground. (TIF) [file pone.0209840.s006.tif]
